# Comparing the Effectiveness of Ultrasonic Instruments Over Manual Instruments for Scaling and Root Planing in Patients With Chronic Periodontitis: A Systematic Review and Meta-Analysis

**DOI:** 10.7759/cureus.31463

**Published:** 2022-11-13

**Authors:** Ranu R Oza, Varsha Sharma, Priyanka Multani, Khushbu Balsara, Pavan Bajaj, Prasad Dhadse

**Affiliations:** 1 Periodontics and Implantology, Sharad Pawar Dental College and Hospital, Datta Meghe Institute of Medical Sciences, Wardha, IND; 2 Periodontics, Sharad Pawar Dental College and Hospital, Datta Meghe Institute of Medical Sciences, Wardha, IND

**Keywords:** systematic review, manual instruments, ultrasonic instrumentation, root planing, scaling

## Abstract

Periodontal disease is a chronic, complex, and infectious condition that affects the periodontium. Its progressive form can be identified by the loss and destruction of the periodontal ligament and the alveolar bone, respectively. Periodontal disease, one of the most prevalent oral cavity diseases, is responsible for tooth loss. Scaling and root planing (SCRP) is a standard, non-invasive periodontal therapy for treating patients with periodontitis. However, there have also been connections to disputed results. According to reports, SCRP alone is ineffective in removing pathogenic microorganisms and their by-products from periodontal pockets. In light of this, our current study aims to determine if using manual or ultrasonic instruments for SCRP in patients with a clinical diagnosis of chronic periodontitis is preferable.

This systematic evaluation compares the effectiveness of manual and ultrasonic devices for SCRP, a line of therapy for individuals with a clinical diagnosis of chronic periodontitis. The databases searched were Prospero, PubMed, MEDLINE, CENTRAL, ClinicalTrials.gov, and Cochrane Library, which exclusively included English-language papers. The articles were also manually searched for any information missed during the search process.

## Introduction and background

Description of the condition

Periodontal disease is a chronic, multifactorial, infectious, and inflammatory disease of the periodontium [1]. Oral bacteria are responsible for the multifactorial expression of periodontitis. According to the most recent research, the host's immune inflammatory response may also play a vital role in destroying the attachment apparatus, which sets off a chain of events that leads to the loss of connective tissue and alveolar bone [2]. According to statistical data, periodontal disease is listed as the sixth most prevalent disease in the world [3]. Therefore, a clinician needs to have information on the disease, its prevalence, and its course to plan activities before designing a course of treatment and care for patients with periodontitis [3].

Numerous environmental and acquired risk factors influence the host response and patient susceptibility to periodontitis, the development of the condition, rate of progression, severity, and responsiveness to treatment. They play a crucial part in formulating the treatment plan and the maintenance therapy. Heredity, smoking while pregnant, menopause (hormonal changes), stress, nutritional deficiencies, systemic diseases like diabetes mellitus, osteoporosis, HIV, neutropenia, syndromes like Marfan's and Ehlers-Danlos syndromes, and many drugs are among these factors: Anti-convulsants, calcium ion (Ca2+) channel blockers, and immunomodulatory substances affect periodontium in one way or the other [4,5]. As a result, it is difficult for a physician to lay up a treatment plan and maintenance therapy because removing bacterial microorganisms from the periodontal pocket is a laborious process that stops and starts again in the oral microbiome. The first stage of the disease, known as gingivitis, where the inflammation solely affects the gingiva, marks the beginning of the illness. Therefore, the type of the disease and its stage determine the treatment strategy and maintenance therapy to be used [6,7].

Description of the intervention

It is now widely acknowledged that the pathophysiology of periodontal disorders involves a complex system. Different groups of people have devised several new methods to quantify periodontal illness. Numerous studies have established a direct connection between advanced non-modifiable risk factors and modifiable risk variables and periodontitis [8]. As was already mentioned, this interventional approach aims to treat periodontal disease to achieve a healthy and functional dentition [9]. The basic occurrences of the disease are frequently addressed by oral hygiene techniques such as appropriate tooth brushing, flossing, sub and supragingival irrigation, anti-biotherapeutics, mechanical therapy, ultrasonic debridement, scaling and root planing (SCRP), and full mouth disinfection [1]. Because SCRP lowers the percentage of pathogenic microorganisms in the periodontal pocket, which is also thought to be the treatment for gingivitis, it has become the gold standard of non-surgical treatment. Numerous clinical studies demonstrate that SCRP aids in reducing clinical features such as clinical attachment loss (CAL), bleeding on probing (BOP), and probing pocket depth (PPD) [10]. Hand instruments such as "curettes, hoes, and scalers" have been used for sub-gingival purposes [11]. Due to the variety in tooth morphology, hand SCRP can be tedious and time-consuming. Thus, when working on patients with deep pockets, it becomes challenging for the physician to insert the hand instruments onto the tooth surface, making sub-gingival debridement a crucial task [12,13]. It led to the development of the ultrasonic instrumentation era. According to the literature, these devices have excellent qualities that enable clinicians to access the furcation area effectively. They have also increased accessibility to reach the depth of pockets without exerting themselves [14]. Numerous research has compared the effectiveness of hand instruments with ultrasonic instruments; some contend that ultrasonic scaling is more effective at achieving microbiological and clinical criteria, while others claim that they are more equivalent to manual SCRP [9,10]. However, there is little information regarding clinical effectiveness and safety. Clinicians typically favor power-driven ultrasonic mechanical tools for sub-gingival debridement. To determine whether ultrasonic instrumentation for SCRP is superior to manual instrumentation in patients with chronic periodontitis, we planned this systematic review [11]. 

Overview of how the intervention might work

The aim of SCRP is to eliminate dental calculus and plaque because they contain bacteria that release toxins that affect the gingiva and periodontal attachment [15]. Scaling is a mechanical periodontal therapy where plaque and calculus are removed utilizing supra and subgingival tooth planes. Cementum and submerged calculus are removed from root surfaces using the root planing procedure to create a smooth and polished surface. In order to evaluate the clinical effectiveness of manual and ultrasonic SCRP for periodontal health in patients with chronic periodontitis, our study used parameters such as BOP, PPD, CAL, and gingival recession (GR). The aim of the study was to evaluate the results of split-mouth designs for randomized controlled trials (RCTs) with open or blinded outcome assessment.

Why it is essential to do this review 

When harmful bacteria and hard tartar deposits called calculus build up on teeth and around the gums, they release toxins that lead to the gum disease known as gingivitis and eventually the destruction of the periodontium, also known as periodontitis. Unfortunately, the buildup occurs in places where conventional dental cleaning, flossing, or even brushing are ineffective. If periodontitis develops over time, a series of events, including inflammation, bleeding, gingival recession, and receding bone tissue, result. The SCRP is done using a hand scaler and an ultrasonic cleaner which vibrates at high frequencies to remove calculus from the teeth. Only a small body of research supports the idea that ultrasonic scaling is more effective than manual scaling. On the other hand, numerous studies claim that ultrasonic and manual scaling results are comparable. Our study aims to compare the effectiveness of machine-driven equipment vs. manual instruments for SCRP. The protocol of this systematic review has been published in the Journal of Critical Reviews (JCR), Volume 6, Issue 6. 

## Review

Methodology

We looked for any ongoing Prospero reviews in our preparation schedule. We also searched PubMed, MEDLINE, CENTRAL, and Cochrane Library databases. We also checked ClinicalTrials.gov to see if there were any registered trials. A search was conducted using medical subject headings (MeSH), their equivalents, and text keywords. We included patients, regardless of age, sex, or race, with a clinical diagnosis of persistent periodontitis who are systemically healthy.

Types of outcome measures

Primary outcomes (which were the main outcomes evaluated when generating conclusions) and secondary outcomes (which contributed to forming findings from the primary outcomes) were the two categories used to describe the aftermath. We used a fixed reference point to measure the distance from the cementoenamel junction to the base of the pocket. The PPD involved the length of the gingival sulcus or pocket from the gingival edge. For BOP, a periodontal probe was introduced into the sulcus at the mesial aspect of the papilla, moved coronally, and then removed. This was repeated on the distal portion of the papilla. The plaque index (PI) is a measure of dental plaque found in the regions along the gingival border used to estimate the state of oral hygiene. Marginal gingiva displacement is apical to the cementoenamel junction.

Data collection and analysis

Three reviewers (KB, PM, and VS) chose the studies based on the selection criteria from the Rayyan online screening tool [16]. We analyzed the papers, titles, and abstracts. Following the inclusion procedure, the full texts of these studies were acquired and reviewed to gather the necessary information. The fourth and fifth reviewers worked together to resolve any issues during the inclusion and exclusion of investigations and any conflicts that arose throughout the inclusion process (RO, PB). Any disagreement was solved by the sixth reviewer (PB). A Preferred Reporting Items for Systematic Reviews and Meta-Analyses (PRISMA) flow chart of all the identified studies was added [17] as advised in part 2, section 11.2.1 of the Cochrane Handbook for Systematic Reviews of Interventions [18]. 

Data extraction and management

Three review authors (RO, KB, and PM) collected data from the studies and presented it in a table titled Characteristics of Studies using a pre-defined data extraction method. Data extraction was followed based on the study's design, participant information, intervention information, and reported results. We invited the fourth reviewer to settle the disagreement amongst the principal reviewers (RO, VS). The fifth reviewer (PD) corrected the disparity in the risk of bias assessment. 

Assessment of risk of bias 

Three reviewers (KB, PM, and RO) used the Cochrane domain-based, two-part tool, as outlined in Chapter 8 of the Cochrane Handbook for Systematic Reviews of Interventions (Higgins 2011), to assess the risk of bias (RoB) of each included study. The disagreement between the primary reviewers was addressed by the fifth reviewer (PB). Following these stages, the RoB was assessed. Sequencing, hiding allocations, blinding participants and staff, blinding outcome evaluation, insufficient outcome information, selective outcome reporting, and other biases, like baseline imbalance.

Data synthesis 

Our aim for starting a meta-analysis was only if the participants, interventions, comparisons, and results of the included studies were conclusive enough on the similarity index to disclose solutions with clinical value and relevance. We used the Cochrane Collaboration's RevMan 2014 statistical program to conduct the meta-analysis. If statistical heterogeneity with an I^2 ^of 50% was found, a random-effects model meta-analysis was then performed.

Measures of treatment effects

After six months post-treatment, we calculated mean differences (MDs) and 95% confidence intervals (CIs) for continuous conclusions and data using mean and standard deviation. We conducted subgroup analysis and heterogeneity research based on the type and length of the intervention provided. In parallel group RCTs, we used individuals as the analytical unit. Elbourne's suggested methodology and meta-analysis include the cross-over designed trials [19]. We included trials by measuring the relevant experimental and control intervention periods. We studied clinical trials under the presumption that it was a parallel group trial of intervention vs. control.

Dealing with missing data 

If the published data was found to be lacking, partial, or inconsistent with RCT protocols, further information was requested from the authors or manufacturers. We aimed to carry out the objective to treat analyses according to the number of studies available. If the chosen studies did not report on the outcome measures of interest, a description of randomization and intent to treat analyses, or had missing data in the study result, the authors and the manufacturers were contacted via email.

Assessment of heterogeneity 

The chi2 test ("P value 0.10 for statistical significance") was utilized, and the I^2^ statistic was used to measure the degree of heterogeneity among the included research results. Heterogeneity was considered significant if I^2^ was more excellent than 75%. Heterogeneity was classified as considerable if it ranged between 50% and 90%. Heterogeneity was deemed moderate if it ranged between 30 and 60%. Heterogeneity was minimal if it was lower than 40% [20]. If statistical heterogeneity exists with an I^2^ of 50%, then potential sources were prospected using prespecified subgroup analysis, and a random-effects model was used and reported. 

Results

**Figure 1:**
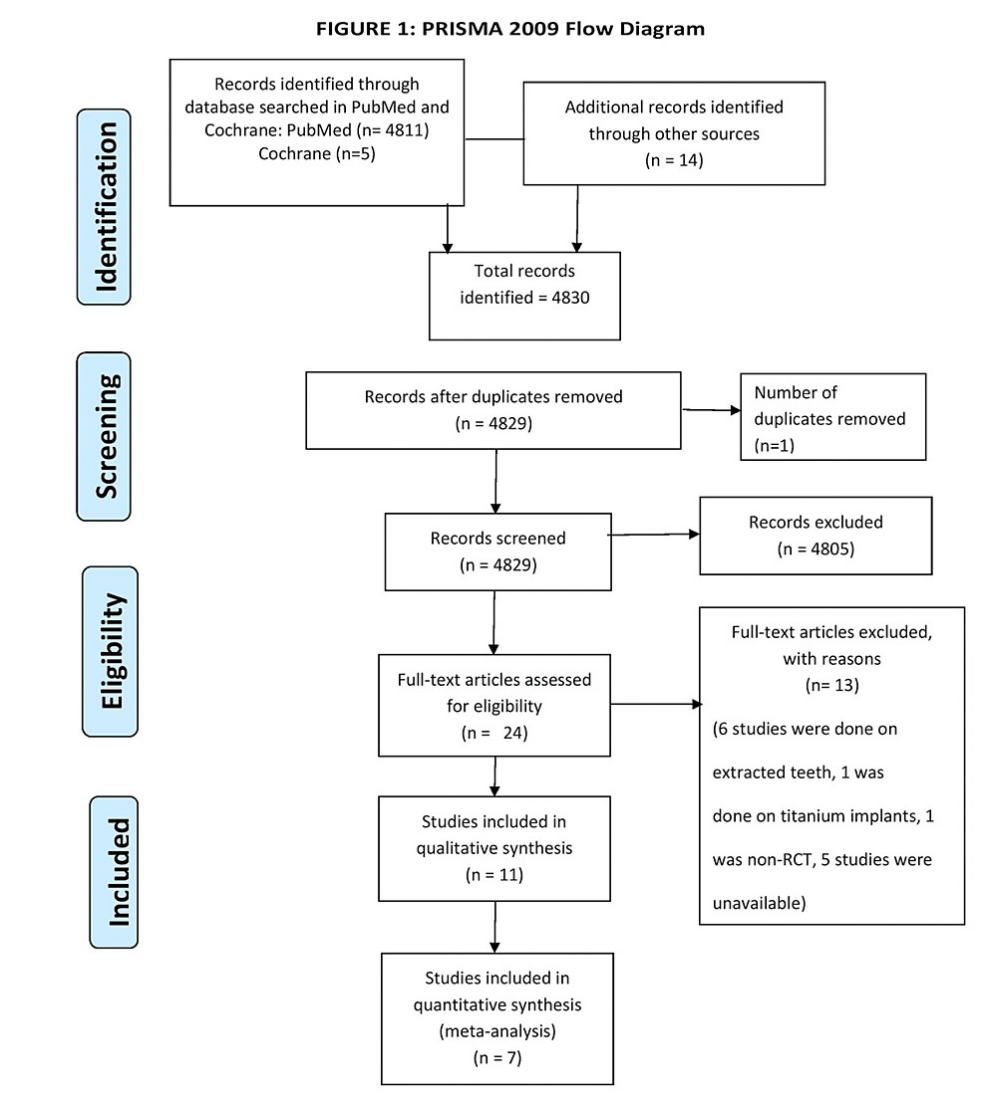
PRISMA flow chart PRISMA: Preferred Reporting Items for Systematic Reviews and Meta-Analyses

Electronic searches turned up 4830 studies in total, including 4816 records from the Cochrane and PubMed databases and 14 entries from other sources. After removing duplicates, 4829 records were screened in total. Of these, 4805 were rejected as they did not follow our inclusion criteria. After retrieving the full texts of the 24 records, we eliminated 13 studies. A total of 11 studies were included for qualitative analysis and have also been mentioned in the summary of the articles. Amongst these 11 studies, we have included quantitative analysis. Figure 1 shows a flow diagram that summarizes the study identification procedure.

Included Studies

We found eleven studies with inclusion and exclusion criteria which were included for qualitative analysis. Of these 11 studies, we selected seven studies for quantitative analysis (meta-analysis). In summaries of papers, the study's specifics are provided (Table 1). We included only English-language articles. Table 1 contains information about the participant recruiting. All trials that had subjects with chronic periodontitis were taken into consideration.

**Table 1:** Summary of articles NR: Not reported, NA: Not applicable, IG: Intervention group, CG: Control group, UD: Ultrasonic-driven, GI: Gingival Index, PI: Plaque index, GBI: Gingival bleeding index, BOP: Bleeding on probing, SCRP: Scaling and root planing, PPD: Probing pocket depth, GR: Gingival recession, CAL: Clinical attachment loss, FMPS: Full mouth plaque score, RAL: Relative attachment level, RD: Recession depth, KTH: Height of keratinized tissue, PBI: Papillary bleeding index, PAL: Probing attachment level, NDP: Number of deep pockets, NMP: Number of moderate pockets, RI: Retention index, CFM: Crevicular fluid measurement

Articles	Country	Type of study	Number of groups	Number of patients	Number of patients who completed the study	Age	Gender (M/F)	Type of periodontal disease	Criteria	Type of intervention	Frequency	Duration	Outcomes	Brushing technique	Instruments used	Time of reporting
Ioannou et al. 2009 [19]	Thessaloniki, Greece	RCT	Two	Group1- 20(SCRP), Group2- 20 (UD)	Group1- 16(SCRP), Group2- 17 (UD)	18-70 years of age	NR	Chronic Periodontitis	PPD>5mm, BOP	SCRP + oral hygiene instructions	Weekly intervals, in 3 to 4 sessions	6 months	Primary-CAL, Secondary-PPD, GBI, PI, microbiological examination	Modified Bass Technique+ Interdental brush	1) Periodontal curettes (Hu-Friedy Gracey Standard Curettes SG 3/4, 11/12, 13/14, Hu-Friedy; 2) Piezoelectric ultrasonic device (EMS Piezons, EMS, Nyon, Switzerland)	6 months
Sculean et al. 2004 [20]	Frankfurt, Germany	RCT	Two	Group1- 19 VUS (10F,9M), Group2- 19 SCRP (11F/ 8M)	Group1- 19 VUS (10F,9M), Group2- 19 SCRP (11F/ 8M)	Mean age: 54years	14 Males/24 Females	Advanced Chronic Periodontitis	Mean PI<1	SCRP + oral hygiene instructions	Baseline, 4, 8, 12, 16, 20, 24 weeks after treatment	6 months	FMPS, PD, GR, CAL, BOP	NA	1)SCRP (Gracey's Curettes, Hu-Friedy Co., Chicago, IL, USA); 2) Vector Ultrasonic System (Durr Dental, Bietigheim-Bissingen, Germany) using straight and curved metal curettes; 3) manual periodontal probe	6 months
Obeid et al. 2004 [21]	Brussels, Belgium	RCT	NA	20	20	40-69 years of age	10 Males/10 Females	Generalized Moderate-to-Severe Adult Periodontitis	PD>4mm	SCRP + oral hygiene instructions	1-week intervals	6 months	PI, PBI, PPD, PAL, NDPs, NMPs	Bass Technique+ Interdental brushing	1) Hand instruments (Ceramicolor, Ash, Dentsply, PA, USA), Sickle scalers, CK6 & 204S, universal curettes, hoes and Hirschfeld files, Perioplaner, Periopolisher; 2)Ultrasonic scaler (universal insert no.1 with the Suprasson-P500 handle, Stalecc, Bordeaux, France)	6 months
Alves et al. 2005 [22]	Sao Paulo, Brazil	RCT	Two	US Group- 6, CC Group-6	US Group- 6, CC Group-6	35-65years	6 Males/6 Females	Moderate-Chronic Periodontitis	Minimum 3 periodontal pockets on incisors, canines, PD:3.5-6.5mm	SCRP + oral hygiene instructions	Baseline+SCRP (before and immediately after)	NA	RAL	NA	1) 5-6 Gracey curettes (Hu-Friedy, Chicago, IL, USA); 2) Ultrasonic scaler (9 N tip and medium intensity), Profi II Ceramic, Dabi Atlante, Ribeirao Preto, SP, Brazil); 3) Computerized electronic probe (Florida Probe, Florida Probe Corporation, Gainesville, FL, USA),	NA
Beuchat et al. 2001 [23]	Zurich, Switzerland	RCT	NA	15 patients	11 patients	NA	NR	Generalized & Localized Adult Periodontitis	PPD>=5mm interproximally	SCRP + oral hygiene instructions	Baseline, 2 months	2 months	PI (O’Leary), BOP, PPD, REC, CAL	Circular technique + Interdental brushing	1) Hand curettes (Gracey and M 23 A, Deppeler SA, Rolle, CH); 2)Periosonic instrument 1 & 2	2 months
Zucchelli et al. 2009 [24]	Bologna, Italy	RCT	NA	11 patients	11 patients	18-40 years	4 Males/7 Females	Chronic Periodontitis	Miller Class I isolated recession defects KTH>1mm apical to root exposure	SCRP + oral hygiene instructions	Baseline, 6 months	6 months	RD, PD, CAL, KTH	Coronally directed roll technique	1) Periodontal curettes; 2) Ultrasonic; 3) Periodontal pressure-sensitive probe	6 Months
Pons-Vicente et al. 2008 [25]	Barcelona, Spain	RCT	Two	Experimental group: 17, Control group: 13	Experimental group: 17, Control group: 13	19-52 years	14 Males/29 Females	Periodontal defects on the distal aspect of the lower 3rd molar	Mesioangular position (partially or totally impacted) 3rd molar	SCRP + oral hygiene instructions	Baseline, 3 months, 6 months	6 months	Pre/postop intrabony defect Pocket depth	Surgical brush + Rinse with 0.12% Chx post-surgery	1) Periodontal curettes (Lucas Curette n° 85; Kohler; Neuhausen ob Eck, Germany); 2) Ultrasonic device, (P5)	6 Months
Walsh et al. 1975 [26]	London, United Kingdom	RCT	NA	15 patients	15 patients	26-44 years	NR	NA	Mild to Moderate Periodontitis	SCRP + oral hygiene instructions	Baseline, 1 month, 2 month	2 months	PI, GI, RI, CFM	NA	1) Hand curette; 2) Ultrasonic scaler ( curette-shaped tip with circular cross-section and rounded end (No. P. 10); 3) Periodontal probe	2 months
Copulos et al. 1993 [27]	Florida, United States	RCT	NA	9 patients	9 patients	32-73 years	7 Males/2 Females	Periodontal disease	History of periodontal disease, active periodontal therapy, and subsequent regular SPT for at least 1 year; 10 sites with PPD ≥ 3.0mm	SCRP + oral hygiene instructions	Baseline, 3 months, 6 months	6 months	PI, GI, PPD, BOP, CAL	NA	1) Gracey curettes; 2)Ultrasonic scalers with modified; 3) electronic probe (standardized force probe set at 25g)	6 months
Christgau et al. 2006 [28]	Dusseldorf, Germany	RCT	NA	20 patients	20 patients	46 years	6 Males/14 Females	Generalized Moderate to Progressive Chronic Periodontitis	At least 4 teeth per quadrant with a PPD=4mm	SCRP + oral hygiene instructions	Baseline, 0.5 month, 1 month, 6 months	6 months	PPD, GR, CAL, BOP	NA	1) Hand curettes	6 months
Christgau et al. 2007 [29]	Dusseldorf, Germany	RCT	NA	20 patients	19 patients	44 years	15 Males/5 Females	Generalized Moderate to Severe Chronic Periodontitis	At least 4 teeth per quadrant with a PPD=4mm	SCRP + oral hygiene instructions	Baseline, 1month, 6 months	6 months	PPD, CAL, BOP	NA	1) Gracey curettes #1/2, #7/8, #11/12, #13/14, Hu-Friedy, Chicago, IL, USA, Dental Explorer (CH3, Hu-Friedy); 2) New Vector Ultrasonic System (Durr, Bietigheim-Bissingen, Germany)	6 months

Intervention

The chosen studies assessed changes in the clinical periodontal parameters following hand scaling, root planing, and ultrasonic scaling, which were divided into distinct groups: a control group and an interventional group. Patients were randomly divided into two groups in each study, with the interventional group receiving ultrasonic SCRP and the control group receiving hand SCRP. The intervention type in each study was SCRP together with oral hygiene instructions.

Comparison

The intervention was SCRP and oral hygiene instructions in all the selected studies. However, the intervention duration and frequency varied in most studies. In most studies, the intervention time was six months [21-27]. Two studies had an intervention duration of two months [27,28]. At the same time, one trial performed SCRP only once in the control and the intervention group [28]. In two studies the frequency of intervention was at baseline, three months, and six months [24,25]; one kept the intervention frequency at baseline, one month, and two months [28], and the other intervened at the frequency of baseline and two months [28]. One study had the frequency of baseline, 0.5 months, one month, and six months, whereas another included study had the frequency of baseline, one month, and six months [26]. One trial presented the intervening frequency at baseline and six months [26]; another had the frequency of baseline, 4, 8, 12, 16, 20, and 24 weeks and it was at weekly intervals in three to four sessions in the study by Ioannou et al. [21,27]. All the studies used periodontal curettes for manual scaling and root planning. In contrast, ultrasonic scalers were used in all the studies for ultrasonic scaling and root planing, except for a few studies where they used ultrasonic scalers [23,24,27,28]. One trial used a piezoelectric ultrasonic device was used [27]; another used Vector Ultrasonic System (Durr Dental, Bietigheim-Bissingen, Germany) [21]; Beuchat et al. used periosonic instruments 1 and 2 [28]; a modified sonic scaler was used by Christgau et al. [26]. All the studies performed the intervention under local anesthesia.

Outcomes

Various parameters were recorded to check the clinical efficacy of SCRP, and one of the factors, periodontal pocket depth (PPD), was recorded in all the studies except two studies [27,28]. The CAL was evaluated [21,22,26,27]. The clinical parameter, relative attachment loss (RAL), was recorded from the margin of a restoration or a stent. The RAL is generally recorded when the CEJ is not present due to restoration or is difficult to determine. The BOP was also assessed. The PI was evaluated in all the studies, including the rate of plaque formation on the tooth surface. Gingival recession (GR) was measured as displacement of the marginal tissue apical to the cementoenamel junction [21,22,24,27,28].

Forest plots

The PPD and CAL were evaluated separately in the interventional (hand SCRP) and control groups (ultrasonic SCRP). All the included studies compared the effectiveness of ultrasonic instruments over manual instruments for SCRP in improving the periodontal parameters. Still, only six studies were eligible for meta-analysis of PPD [22-24,26-28], and five were eligible for meta-analysis of CAL [22-24,27,28]. Overall effect size estimates are from a meta-analysis. 

Probing Pocket Depth (PPD)

**Figure 2:**
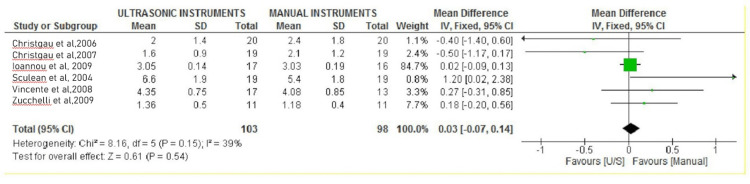
Forest plot depicting probing pocket depth Figure created via Cochrane's RevMan 2014 for six studies [28,29,19,20,25,26]

All six studies used periodontal curettes for manual scaling and root planning, whereas ultrasonic scalers were used in all the studies for ultrasonic scaling and root planning. Most of the studies by Christgau et al. in 2006 and Christgau et al. in 2007 favored the efficacy of ultrasonic instruments over manual instruments for SCRP in terms of probing depth outcomes [24]. One study showed no significant difference in the efficacy of ultrasonic and manual instrumentation [27]. Studies have also recorded an improved reduction in probing depth on scaling and root planning by manual instrumentation over ultrasonic instrumentation [21,25]. Different probing depths were recorded for single-rooted and multi-rooted teeth with the initial pocket depth of 4mm to 5mm and >6mm each [21]. We considered the data for single-rooted teeth with >6mm of initial pocket depth. This data showed significant results favoring manual instrumentation over ultrasonic instrumentation for scaling and root planning. The overall meta-analysis showed no significant reduction in PPD six months after SCRP using ultrasonic instruments over manual instruments (MD 0.03; 95% CI 0.07 to 0.14; P=0.15; I2 39%; six studies; 151 participants). The probing pocket depth analysis is mentioned in Figure 2.

Clinical Attachment Loss (CAL)

**Figure 3:**
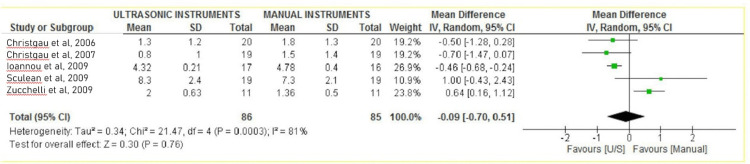
Forest plot depicting clinical attachment loss Figure created via Cochrane's RevMan 2014 for five studies [28,29,19,20,24]

All five studies used periodontal curettes for manual scaling and root planning, whereas ultrasonic scalers were used in all the studies for ultrasonic scaling and root planning. CAL gain favored the intervention group, i.e., the ultrasonic SCRP group, over manual instrumentation [26,27]. Two studies showed improved CAL outcomes with manual instrumentation over ultrasonic [21,23]. The overall meta-analysis showed no significant reduction in CAL six months after SCRP using ultrasonic instruments over manual instruments (MD -0.09; 95% CI -0.70 to 0.51; P =0.0003; I2 81%; five studies; 121 participants). The clinical attachment loss analysis is mentioned in Figure 3.

Risk of bias

All studies were low-risk bias studies. The risk of bias in other domains was unclear or low.

Allocation (Selection Bias) Depicting Random Sequence Generation

All studies were at low risk of selection bias for random sequence generation. One RCT randomly assigned patients into two treatment groups using random tables, and another randomly assigned the treatment type and the operated site based on a board of random permutations of 10 elements immediately before the procedure [25,27]. One applied a “split-mouth design where one quadrant of the upper and lower jaws was randomly selected applying a random number table, which was generated using SPSS software version 11.5 and 13.0 (IBM Corp., Armonk, NY, USA),” respectively [26]. Two RCTs mentioned that a random allocation sequence was produced [21,23].

All the included studies reported a low risk of allocation concealment, which was unclear for one study [26]. One included trial used a sealed envelope with a card indicating the treatment [27]. A toss of a coin was the choice of method for allocation to therapy in some studies [21,23]. Others randomly assigned the treatment immediately before the procedure [25]. Treatment allocation was concealed from the operator in one study [26].

Blinding (Performance Bias and Detection Bias)

Most studies were double-blinded [23,27]. Two studies were single-blinded, with one masked for only the operator [25]. Some studies were blinded for the examiner [21,26].

Incomplete Outcome Data (Attrition Bias)

All the studies reported low attrition bias except for one study which recorded a loss to follow-up of 17 patients in the experimental group and 13 patients in the control group and, thus, was judged as having high-risk attrition bias [25].

Selective Reporting (Reporting Bias)

**Figure 4:**
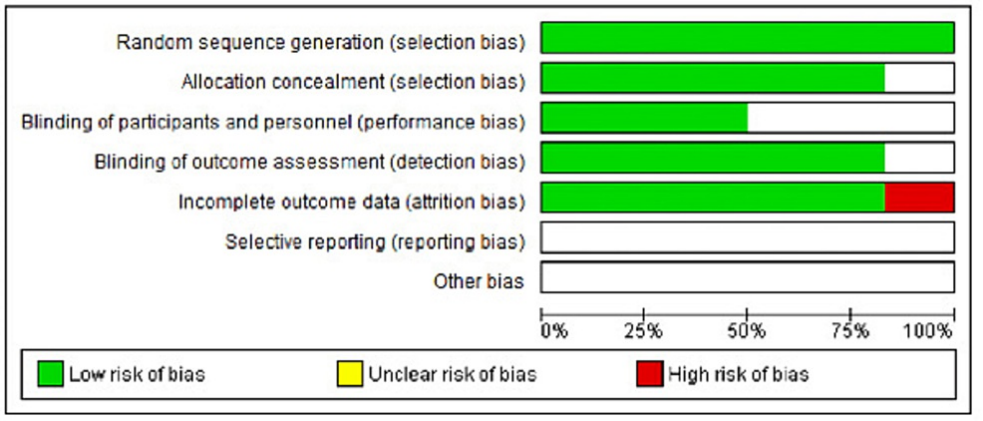
Risk of bias Graph A review of authors' judgments about each risk of bias item presented as percentages across all included studies. Figure created via Cochrane's RevMan 2014

**Figure 5:**
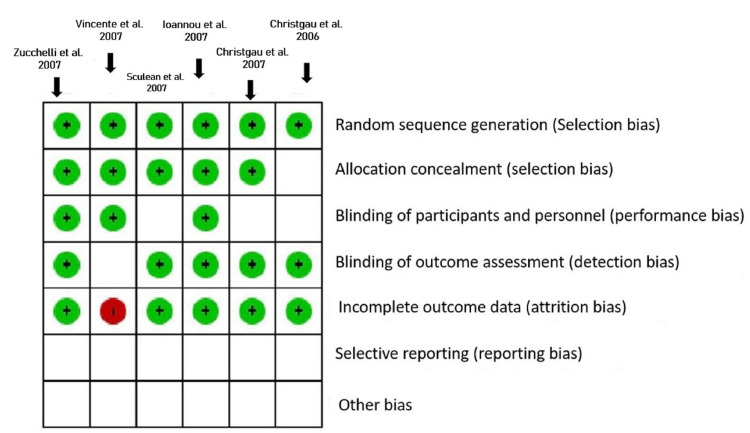
Risk of bias summary A review of authors' judgments about each risk of bias item for each included study [24,25,20,19,28,29] Figure created via Cochrane's RevMan 2014

None of the included studies have their protocols registered. Hence they have been judged unclear. The risk of bias is shown in Figures 4, 5.

Discussion 

We included 11 studies reporting data from 218 participants aged 18 to 70 years, comparing the effectiveness of ultrasonic instruments over manual instruments for SCRP. Studies reported data on periodontal parameters such as PPD, CAL, and BOP. According to the results, it cannot be concluded whether or not ultrasonic SCRP is more effective than manual SCRP based on PPD, CAL, and BOP as outcome variables. Due to the differences in the time of reporting of the included studies, they were not fit for meta-analysis [26-28]. We had only those studies with continuous data outcomes for meta-analysis, and therefore, some studies were excluded as they were reported as dichotomous data types [22,24]. Meta-analysis for pocket depth measurement was done including six studies. Meta-analysis for CAL was done including five studies [21,23,25-27].

Overall Completeness and Applicability of Evidence

This review is focused on the effectiveness of ultrasonic versus manual scaling and root planning in improving periodontal parameters in patients with chronic periodontitis. We found that 11 studies met our inclusion criteria. These studies are mentioned in the summary of the articles table. Out of these, six were included in a meta-analysis of PPD and five in a meta-analysis of CAL. This review does not cover these outcomes: gingival index, plaque index, and gingival recession. Clinicians should understand that we only assessed the efficacy of ultrasonic and manual instruments for scaling and root planning in patients with chronic periodontitis for six months.

Quality of the Evidence

The quality of the evidence is good due to the low risk of bias and precision of the results of the included studies (summary of findings for the main comparison). It can be attributed to the fact that the included studies were blinded. The protocol for this review went through a minor change (differences between protocol and review). The protocol included a PI as a secondary outcome. The change may be justified but could still be a source of bias in the review process.

## Conclusions

It is advisable to remove the deposits that have accumulated on the teeth by scaling and root planing. Using a hand scaler or an ultrasonic scaler, calculus and bacterial deposits are removed subgingivally. To remove calculus from the teeth, an ultrasonic scaler uses high frequency. The concept that ultrasonic scaling is more efficient than manual scaling is only partially supported by the research. We discovered no appreciable difference in the efficacy of hand instruments and ultrasonic/sonic equipment while treating chronic periodontitis through this meta-analysis. The occurrence and severity of side effects following the two treatment regimens did not significantly differ, despite the lack of evidence to the contrary. However, a small amount of research suggests that manual instrumentation is superior to ultrasonic equipment in terms of effectiveness. Only a few studies have found ultrasonic instrumentation to be superior. Meta-analysis findings, however, do not support a statistically significant distinction between the two therapy regimens. We conclude from this comprehensive analysis that high-quality controlled RCTs are necessary to evaluate the clinical efficacy of ultrasonic/sonic scaling. To determine whether different subgingival debridement techniques are cost-effective, it is crucial to evaluate the progression of periodontal disease or the survival of teeth as the main outcome variables as well as biopsychosocial components (such as comfort and aesthetics), negative effects (such as root sensitivity and pain), and the operator's health and safety. By taking into account the Consolidated Standards of Reporting Trials (CONSORT) principles when conducting (planning and reporting) investigations, researchers should concentrate on improving study quality as this will help provide accurate data for the conclusion and facilitate evaluation after the publication of these studies. 
